# Acute Stroke Management: Overview and Recent Updates

**DOI:** 10.14336/AD.2021.0311

**Published:** 2021-07-01

**Authors:** Mary Hollist, Larry Morgan, Rainier Cabatbat, Katherine Au, Maaida F Kirmani, Batool F Kirmani

**Affiliations:** ^1^Memorial Healthcare Institute for Neurosciences, Owosso, MI, USA.; ^2^Bronson Neuroscience Center, Kalamazoo, MI, USA.; ^3^George Washington University, School of Medicine & Health Sciences, Washington DC, USA.; ^4^School of Public Health Texas A&M University, College Station, TX, USA.; ^5^Texas A&M University College of Medicine, College Station, TX, USA.; ^6^Endovascular Therapy & Interventional Stroke Program, Department of Neurology, CHI St. Joseph Health, Bryan, TX, USA.

**Keywords:** Intravenous tissue plasminogen activator (IV tPA), mechanical thrombectomy, tenecteplase, stroke, thrombolytics

## Abstract

Stroke is a leading cause of morbidity and mortality in the United States. Whether hemorrhagic or ischemic, stroke leads to severe long-term disability. Prior to the mid-1990s, the treatment offered to a patient who presented with an acute stroke was mainly limited to antiplatelets. The lack of adequate treatment, in particular, one without reperfusion contributed to the disability that ensued. There have been many advances in stroke care within the past two decades, especially with the acute management of ischemic stroke. Even with these advances, it is quite alarming that only a fraction of patients receives acute stroke treatment. Numerous trials were conducted to broaden treatment eligibility in hopes that more patients can be treated acutely and safely. These trials have tested both the time window for IV tPA and endovascular therapy (EVT). Acute stroke management is moving from a universal time window approach to a concept of tissue preservation. Specifically, preserving cerebral blood flow, the penumbra, and reducing the risk of a second event. This movement is being executed through the use of multimodal CT and MRI, as well as individualizing treatment to our patients. Minimizing the initial effect of stroke changes the outcome and leads to an increased likelihood of functional independence. In this review, we discuss the recent updates of acute ischemic stroke management in regards to mechanical thrombectomy as well as thrombolytics including tenecteplase.

The term stroke was originally referred to as “apoplexy,” after its discovery by Hippocrates over 2500 years ago. The term “apoplexy” in Greek means “struck by violence” [1]. This signifies its sudden onset of symptoms, mainly paralysis, as being a sudden strike upon one’s physical wellness. Overtime, the term has evolved from its archaic origins to a form of brain attack. This change in terminology conveys the wide array of knowledge gained about the disease process as more information is being revealed about its pathophysiology and clinical course. In the last couple of decades, there have been numerous advancements in ischemic stroke care not only with thrombolytics, but also with endovascular therapy. Both treatments have revolutionized the acute management of ischemic stroke. Despite this, stroke remains a leading cause of disability worldwide, and to date, is the fifth leading cause of death in the United States [2]. This signifies the impact and burden stroke has on our society. The care we provide in the acute setting, is aimed at reducing this burden and minimizing disability. It is apparent how dynamic the management has been with the recent trials conducted as summarized in Figure 1. Such trials will continue to influence and impact the care we provide in hopes of reducing the burden it has in our community.

**Figure 1.**
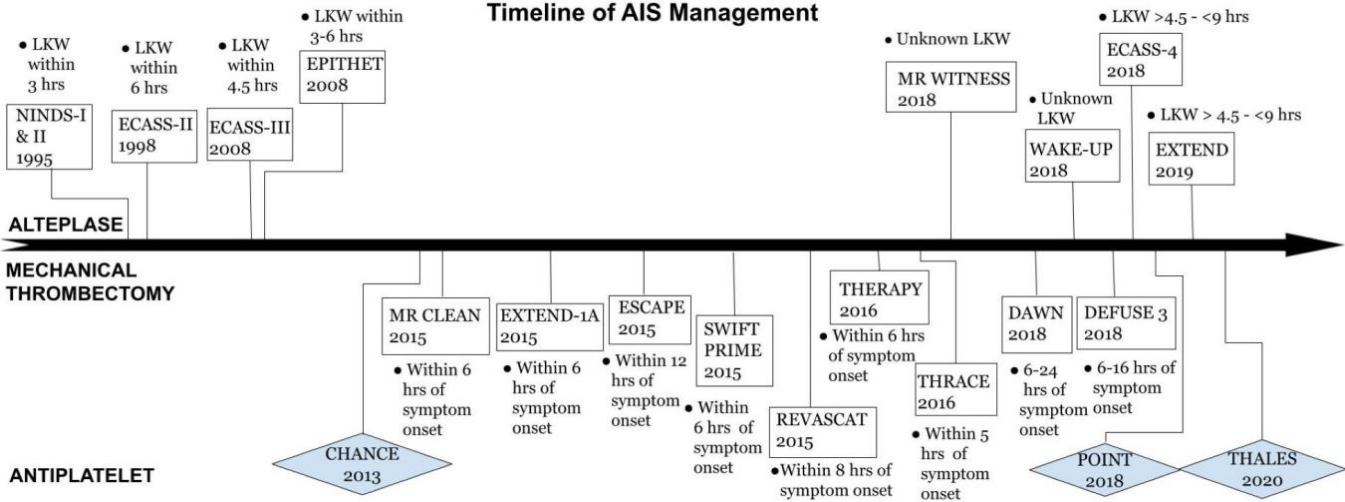
Timeline of AIS Management.

## 1.Thrombolytics

### 1.1 Alteplase

Thrombolytic therapy became the standard of care for acute ischemic stroke after the publication of the Tissue Plasminogen Activator (tPA) for Acute Ischemic Stroke conducted by the National Institute of Neurologic Disorders and Stroke (NINDS tPA trial). The trial demonstrated efficacy of treatment with IV tPA within 3 hours of symptom onset, however, it was associated with a significant risk of symptomatic hemorrhage [3]. Due to the clear benefits of reduced disability and undeniable positive results, the treatment was approved by the Food and Drug Administration (FDA) in 1996. Subsequent studies have been carried out in hopes of safely expanding the use of IV tPA beyond the 3-hour window [4-14]. For example, up to 4.5 hours, 6 hours, 9 hours, and even with wake-up strokes and unknown last well time. Despite the positive outcome of some studies, there are still concerns regarding utilization of tPA in an extended time window. The concerns are based on the risk of hemorrhage without a substantial proven clinical benefit. In addition, there are challenges in determining IV tPA eligibility. Treatment with IV tPA is time sensitive. Determining eligibility in the acute setting can be challenging especially with an inadequate history such as cases involving aphasia or those with fluctuating symptoms. Patients without an established last known well time are typically excluded from treatment. Studies conducted since the NINDS trial have attempted to address these concerns through the utilization of advanced imaging modalities. Such trials are listed in Table 1.

In 2008, the European Cooperative Acute Stroke Study (ECASS) III was the first trial that led to the expansion of the time window from within 3 hours of last known well to 4.5 hours. Using a more stringent criteria, patients treated with IV tPA between 3 to 4.5 hours from last known well were more likely to be functionally independent when compared to placebo. Also, the rate of symptomatic hemorrhage was lower than that reported in the NINDS tPA study [3,5].

In order to further expand the window from the 4.5-hour timeline demonstrated by ECASS III, subsequent studies assessed the use of multimodal imaging techniques in triaging acute ischemic stroke. Computed tomography (CT) of the brain can be obtained rapidly, therefore, it has been the imaging modality of choice for acute ischemic stroke. Though CT is useful in disqualifying patients for thrombolytics by identifying hemorrhage or subacute ischemic strokes, more advanced imaging modalities such as MRI and perfusion studies have demonstrated efficacy in identifying patients that are eligible for treatment outside the traditional treatment window, especially those without a clear last known well time [5-8]. Advanced imaging modalities can be used to evaluate the evolution of an ischemic stroke and assess the safety of thrombolytic therapy in a method that is not solely based on time but based on the concept of a “tissue clock” [6,7,9]. Using this concept, WAKE UP and MR WITNESS trials demonstrated both the efficacy and safety of treating patients with tPA presenting greater than 4.5 hours of last known well by using MRI. In the WAKE-UP trial, patients were eligible if the MRI diffusion weighted imaging identified an area of restricted diffusion with apparent diffusion correlation without a visible lesion on fluid-attenuated inversion recovery (FLAIR). Patients treated with IV tPA demonstrated a significant increase in functional independence when compared to placebo without any significant increase in hemorrhage or mortality [6]. The MR WITNESS study took an additional step of demonstrating the safety of administering IV tPA when an acceptable quantitative measurement of the FLAIR hyperintensity was obtained. In the study, the observed FLAIR hyperintensity in the area of the acute stroke was compared to the corresponding contralateral normal appearing tissue. This measurement obtained is known as the signal intensity ratio (SIR). IV tPA was deemed safe with a SIR value less than 1.15. Safety outcomes in MR WITNESS were comparable to ECASS III [7]. It is important to note that in contrast to WAKE UP, MR WITNESS was designed as a phase II open label safety study and not to demonstrate efficacy.

**Table 1:** Selected Endovascular Therapy Trials for Acute Strokes.

Clinical Trial	Citation	Study Design	Treatment window for Intervention	Number of Patients (n)	Rates of Symptomatic ICH (Treatment versus Control)	Outcome
EXTEND	Ma et al. NEJM 2019 [27]	Multicenter, randomized, placebo-controlled trial	4.5 - 9.0 hours	225 out of 310 planned Trial terminated because of a loss of equipoise; positive result from WAKE-UP trial	6.2% vs 0.9%	Primary outcome of excellent functional outcome at 90 days was not met. However, there was a higher percentage of patients with no or minor neurologic deficits than the use of placebo
WAKE UP	Barow et al. JAMA Neurol. 2019 [28]	Multicenter, double-blind, placebo-controlled randomized clinical trial	Unknown time of symptom onset	503	2.0% vs. 0.4%	Those treated with IV tPA had a favorable outcome at 90 days. No statistically significant difference in regard to symptomatic hemorrhage.
MR.WITNESS	Schwamm et al. Ann Neurol 2018 [7]	Open-label, multicenter, phase 2a study	Unwitnessed onset at 4.5 to 24 hours from last known well	80	1.3% vs. 0%	Primary outcome of safety (symptomatic hemorrhage and edema) was met.
ECASS-4	Amiri et al. Int J of Stroke 2016 [8]	Multicenter, double-blind, placebo-controlled randomized clinical trial	4.5 and 9 hours	119 out of 264 planned Trial terminated because of slow recruitment	1.6%vs. 0%	No significant difference in the modified Rankin scale at day 90. No mortality difference
ECASS-III	Hacke et al. NEJM 2008 [29]	Multicenter, randomized double-blind trial	3-4.5 hours	821	2.4% vs. 0.2%	7.2 % absolute difference in regards to favorable outcome. No significant difference in mortality
EPITHET	Davis et al. Lancet Neurol. 2008 [30]	Multicenter, phase II double-blind, placebo-controlled randomized trial	3-6 hours	101	7.7% vs. 0%	Primary outcome, which was a disease oriented imaging outcome of lower infarct growth was not met. However, IV tPA had a significant increase in reperfusion. No statistical differences in mortality or good functional outcome.
ECASS-II	Hacke et al. Lancet 1998 [4]	Multicenter, randomized double-blind trial	Less than 6 hours	800	8.8% vs. 3.4%	No significant difference in regard to favorable outcome detected at 90 days
NINDS- II	NINDS Study Group NEJM 1995 [3]	Multicenter, randomized double-blind placebo trial	Less than 3 hours	333	6.4%vs. 0.6%	12% absolute increase in patients with little to no disability. Patients treated with IV tPA had significant improvement of mRS at 90 days. No mortality difference
NINDS-I	NINDS Study Group NEJM 1995 [3]	Multicenter, randomized double-blind placebo trial	Less than 3 hours	291	None	No statistically significant difference in improvement of NIHSS at 24 hours

Treatment with IV tPA outside the standard 4.5-hour window was further evaluated by two separate trials, ECASS IV and EXTEND. Both trials assessed the efficacy of IV tPA with perfusion imaging. ECASS IV and EXTEND enrolled patients with a last known well time between 4.5 to 9.0 hours who were not eligible for mechanical thrombectomy, however, with a perfusion to diffusion mismatch ratio of 1.2 or greater [15,16]. Both trials demonstrated a significant rate of symptomatic hemorrhage with IV tPA, but the rates were similar to previous trials such as the NINDS tPA trial and ECASS III [3,4,15,16]. ECASS IV did not demonstrate clinical significance in 90-day outcomes compared to placebo [15]. In contrast, EXTEND demonstrated functional independence at 90 days when adjusted for age and stroke severity (adjusted risk ratio 1.44, p=0.04) with the unadjusted risk ratio trending towards significance prior to early termination of the study [16]. Both studies were terminated prior to completion. ECASS IV’s enrollment declined after publication of the extended window thrombectomy trials, while EXTEND was terminated due to loss of equipoise after publication of the WAKE UP trial [15,16].

With careful patient selection, IV tPA is a safe and effective treatment. Exclusion of those with contraindications to IV tPA is essential given the fine balance that exists with a good favorable outcome and the risk of hemorrhage. Many previous trials excluded patients with mild neurological deficits. The practice of administering IV tPA has remained a gray area in cases of mild non-disabling stroke. The recent PRISMS trial aimed to address this issue and was the first double-blind, multicenter, randomized controlled clinical trial comparing alteplase to aspirin. It tested the efficacy and safety of alteplase administered within 3 hours of symptom onset in patients presenting with minor neurologic deficits [17]. The median NIHSS score in the PRISMS trial was 2, and the median time to treatment was 2.7 hours. Findings from the study demonstrated no significant difference in achieving a favorable neurological outcome at 90 days, but an increased risk for symptomatic intracranial hemorrhage (3.2% with alteplase vs. 0% with aspirin) [17]. Overall, the results indicated that patients with minor non-disabling neurological deficits who were treated with alteplase did not benefit when compared to aspirin. However, it is important to note that symptoms were mild in nature, therefore, participants likely had a higher probability of improvement despite treatment. Also, this trial was terminated early, therefore, no definitive conclusion can be made.

### 1.2 Tenecteplase

Tenecteplase (TNK), a tissue plasminogen activator, is a bioengineered thrombolytic that has a longer half-life and higher affinity to fibrin than alteplase. Currently, TNK is FDA approved for treatment of myocardial ischemia and is under further investigation for treatment of acute ischemic stroke [10,11]. TNK’s biochemical profile has proven practical advantages such as the ability to be given as a single bolus and at a reduced cost [10]. The Norwegian Tenecteplase Stroke Trial (NOR-TEST) was a phase III trial comparing TNK to alteplase within the 4.5-hour window. NOR-TEST demonstrated that TNK was as effective and as safe as alteplase in the treatment of ischemic stroke without any significant difference in functional outcome. Also, there was no significant difference in the rate of symptomatic hemorrhage. Mortality rates in patients with severe strokes, defined by investigators as NIHSS score > 15, were significantly increased in the TNK group compared to alteplase, p=0.045. Similarly, a recent small phase II study tested TNK’s efficacy within the 6-hour time window. In this study, TNK was only administered when a penumbra was observed on CT perfusion. The TNK treated group when compared to the alteplase treated group had significantly better reperfusion and clinical improvement [13]. With regards to only large vessel occlusions, the Tenecteplase versus Alteplase before Endovascular Therapy for Ischemic Stroke (EXTEND-IA TNK) trial enrolled patients presenting within 4.5 hours of symptom onset that were eligible for mechanical thrombectomy. Though the EXTEND-IA TNK was powered as a non-inferiority study, it showed TNK had significantly improved reperfusion rates, favorable 90-day outcomes, and reduced mortality without any significant difference in hemorrhagic events when compared to alteplase [18].

Though this data is promising, TNK is not FDA approved for treatment of acute ischemic stroke. Further randomized trials are ongoing to assess its safety and efficacy. The TIMELESS trial is currently active and still enrolling patients. It started in March 2019 and has an estimated study completion date of April 2022 (ClinicalTrials.gov number, NCT03785678). The goal of this trial is to assess the safety and efficacy of tenecteplase in patients presenting with AIS from 4.5 to 24 hours of symptom onset. Criteria include patients who are not IV tPA eligible but have an LVO as well as a mismatch on their CT perfusion and MRI images. Trials like TIMELESS will provide more information about the safety of expanding the treatment time window for thrombolysis and also aid in providing treatment to a broader range of patients.

## 2.Endovascular

Mechanical thrombectomy (MT) is a fairly new treatment used in the management of acute ischemic stroke. Similar to other developing techniques and treatments, the beginnings were filled with tribulations. Early trials failed to demonstrate MT as a safe or effective treatment. As a result, there were concerns and widespread discussions pertaining to its future. It was unclear if training programs should remain open given its doubts and assumptions of there being a limited role for neurointerventionalists in the future [19]. Not dissimilar to IV thrombolysis, we quickly learned that appropriate patient selection is paramount to establishing evidence in support of its effectiveness. In 2015, multiple large randomized control trials established the effectiveness of MT. The first to do so was MR CLEAN, which was completed in the Netherlands. The protocol called for confirmation of a proximal large vessel occlusion (LVO) within 6 hours of last known well (LKW) utilizing stent-retriever devices along with permitted use of intraarterial thrombolytics. MR CLEAN aimed to assess the efficacy of MT using the modified Rankin Disability Scale (mRS) at 90 days. Patients were randomized to receive either standard care with IV tPA within 4.5 hours or MT with or without IV tPA. Of those enrolled, 89% were treated with IV tPA prior to randomization. In regard to functional independence at 90 days, the results demonstrated an absolute percentage difference of 13.5% with a common odds ratio of 1.67 [95% CI,1.21 to 2.30] [20], proving MT to be safe and effective. The HERMES publication provided a pooled analysis of 1287 participants in the 5 positive trials conducted between 2010 to 2014: MR CLEAN, ESCAPE, REVASCAT, SWIFT PRIME, and EXTEND IA. Overall, the results were astounding. The HERMES meta-analysis showed a widespread improvement in functional outcomes at 90 days with a number to needed treat of 2.6 [21].

The rejuvenated research into MT over the next few years, and supplementary studies continued to provide additional evidence of its safety and efficacy. More recently, the spotlight has focused on advanced imaging with the use of CT perfusion in those presenting with symptoms of acute ischemic stroke beyond 6 hours from their LKW. The DAWN trial required evidence of a proximal LVO on vascular imaging, presentation within a 6-to-24-hour window of LKW and a baseline mRS of 0-1. Participants were then separated into 3 groups based on age, infarct volume, and NIH stroke scale. Inclusion criteria for those under 80 years old required either a NIHSS of 10 or more along with an infarct volume of less than 31mL by automated RAPID AI interpretation. Also, those with a NIHSS of 20 or more and under the age of 80 required an infarct volume between 31 and 51mL. For those older than 80 years, a NIHSS of 10 or more and an infarct volume less than 21mL was required. The primary outcome was utility weighted mRS at 90 days. Enrollment ceased at the prespecified interim analysis due to the positive findings. There was a 2-point difference in the utility weighted mRS at 90 days in favor of MT in those meeting the above-described criteria. It is also important to note that the rates of symptomatic intracerebral hemorrhage did not differ between the groups [22].

DEFUSE 3, another trial completed simultaneously with DAWN, used a slightly different criteria, but showed a similar efficacy for MT. Patients were enrolled if they presented within 6 to 16 hours from LKW with a known anterior circulation LVO, an initial core infarct volume of less than 70mL, and a mismatch ratio between core infarct to penumbral tissue of 1.8 or more. The primary outcome was measured by mRS. Similar to DAWN, the trial was terminated early at the prespecified interim analysis due to a significant positive treatment effect demonstrating a higher likelihood of improvement over standard treatment on mRS at 90 days [23].

With a number needed to treat of 2.8 for DAWN, along with a slightly higher value for DEFUSE 3, both trials expanded the treatment window for acute ischemic stroke treatment. Indisputably, MT is effective even in nonagenarians [22]. Table 2 provides a summarization of pertinent findings of the mechanical thrombectomy trials.

## 3.Non-treatment candidates

Despite the strides made towards the management of acute ischemic stroke, not all patients are treated with thrombolytics or mechanical thrombectomy. This can be explained by certain reasons such as the presence of an underlying contraindication, mild non-disabling symptoms, or delayed presentation out of the treatment window. Those who do not receive acute therapy are often treated with antiplatelets. Although management with IV tPA is superior, prompt initiation of treatment even with antiplatelets minimizes the risk of early stroke recurrence and improves patient outcome. Several trials have explored the combination of dual antiplatelet in this setting in regard to its efficacy with secondary prevention.

The Clopidogrel with Aspirin in Acute Minor Stroke or Transient Ischemic Attack trial, (CHANCE trial), explored the association between dual antiplatelet therapy and the prevention of stroke following a minor stroke or high-risk TIA. Over 5000 participants were enrolled in the study. Patients were randomized into two treatment groups if they presented within the first 24 hours of symptoms onset with a diagnosis of either an acute minor ischemic stroke (defined as NIHSS≤3) or high-risk TIA (ABCD2 score≥4). Both groups received aspirin on the first day with a dose ranging from 75-300 mg. Patients in the dual antiplatelet group had a 300 mg loading dose of clopidogrel on day one. On days 2 to 21, patients in this group received clopidogrel 75 mg and aspirin 75mg daily. On days 22 to 90, these patients received a single antiplatelet agent (clopidogrel 75mg) and aspirin in the form of placebo. The primary efficacy outcome was either a new ischemic or hemorrhagic stroke at the 90-day time point. The primary safety outcome was a new bleeding event (moderate to severe). The primary outcome of a new stroke occurred less in the combination therapy group when compared to the aspirin-only group. Also, treatment with combination therapy was not associated with an increased rate of hemorrhage. Findings from this study found that prompt management with dual antiplatelet reduced the risk of further strokes by 32% with a number needed to treat of 29. It is important to highlight that this study was not an international trial and was only completed in China. The study population had less rates of traditional stroke risk factors like hypertension, hyperlipidemia, and diabetes. Also, the primary stroke mechanism in the Chinese population is intracranial atherosclerosis which differs from other developed countries. A big limitation is that the findings of the CHANCE trial cannot be generalized to other stroke patients worldwide [24].

Due to this, a randomized, multicenter trial was performed. The Clopidogrel and Aspirin in Acute Ischemic Stroke and High-Risk TIA trial (POINT trial), like the CHANCE trial, explored whether clopidogrel and aspirin in combination reduced the rate of recurrent stroke at 3 months after a minor stroke or high-risk TIA. Similar to the CHANCE trial, patients were included if they had a NIHSS score of 3 or less or an ABCD2 score of 4 or more. Unlike the CHANCE trial, patients underwent randomization within 12 hours of symptom onset and received a clopidogrel loading dose of 600 mg. Patients were either assigned to clopidogrel and aspirin or placebo and aspirin in a 1:1 ratio. The primary outcome was a composite of myocardial infarction, ischemic stroke, or death from ischemic vascular causes which occurred less in the combination therapy group. Also, those in the combination therapy group had a lower incidence of stroke, however, a higher risk of hemorrhagic events compared to those who received aspirin only. The trial was terminated in 2017 due to the high rates of major hemorrhage. The POINT trial was an international, multicenter trial; therefore, participants were more diverse, and findings are more generalizable than the CHANCE trial. Patients in the POINT trial received a higher loading dose of clopidogrel and were treated with dual antiplatelet therapy for a longer period. The period of most observed efficacy and benefit from dual antiplatelet therapy was within the first month only. The risk of hemorrhage, however, was also observed in the same time period, and remained persistent throughout the entire trial [25].

The role and benefit of ticagrelor in stroke is not well established. Clopidogrel is a well-studied drug, however, its effectiveness is less certain in some patients due to polymorphism of the CYP2C19 gene. The Ticagrelor and Aspirin or Aspirin Alone in Acute Ischemic Stroke or TIA trial (THALES trial), assessed the efficacy of stroke prevention with combination therapy of ticagrelor and aspirin vs. aspirin only. Patients with mild to moderate noncardioembolic acute ischemic stroke with an NIHSS score of 0-5 or a TIA with ABCD2 of 6 or more were included in the study. Patients received a loading dose of 180mg of ticagrelor, followed by ticagrelor or placebo in two 90 mg tablets at 12-hour intervals throughout the trial period. Concurrently, patients also received a loading dose of aspirin, at a recommended dose of 300-325 mg, followed by a daily dose of 75-100 mg. The primary outcome (composite of stroke or death) occurred at a lesser rate in the group receiving both aspirin and ticagrelor than the aspirin-only group. However, the combination therapy group had more hemorrhagic events. The trial was discontinued due to the bleeding rates in the combination therapy group. The results showed that patients with mild to moderate ischemic stroke or TIA who received both ticagrelor and aspirin within 24 hours of symptom onset had a lower risk of stroke or death at 30 days, however, a significant risk of severe hemorrhage [26].

**Figure 2.**
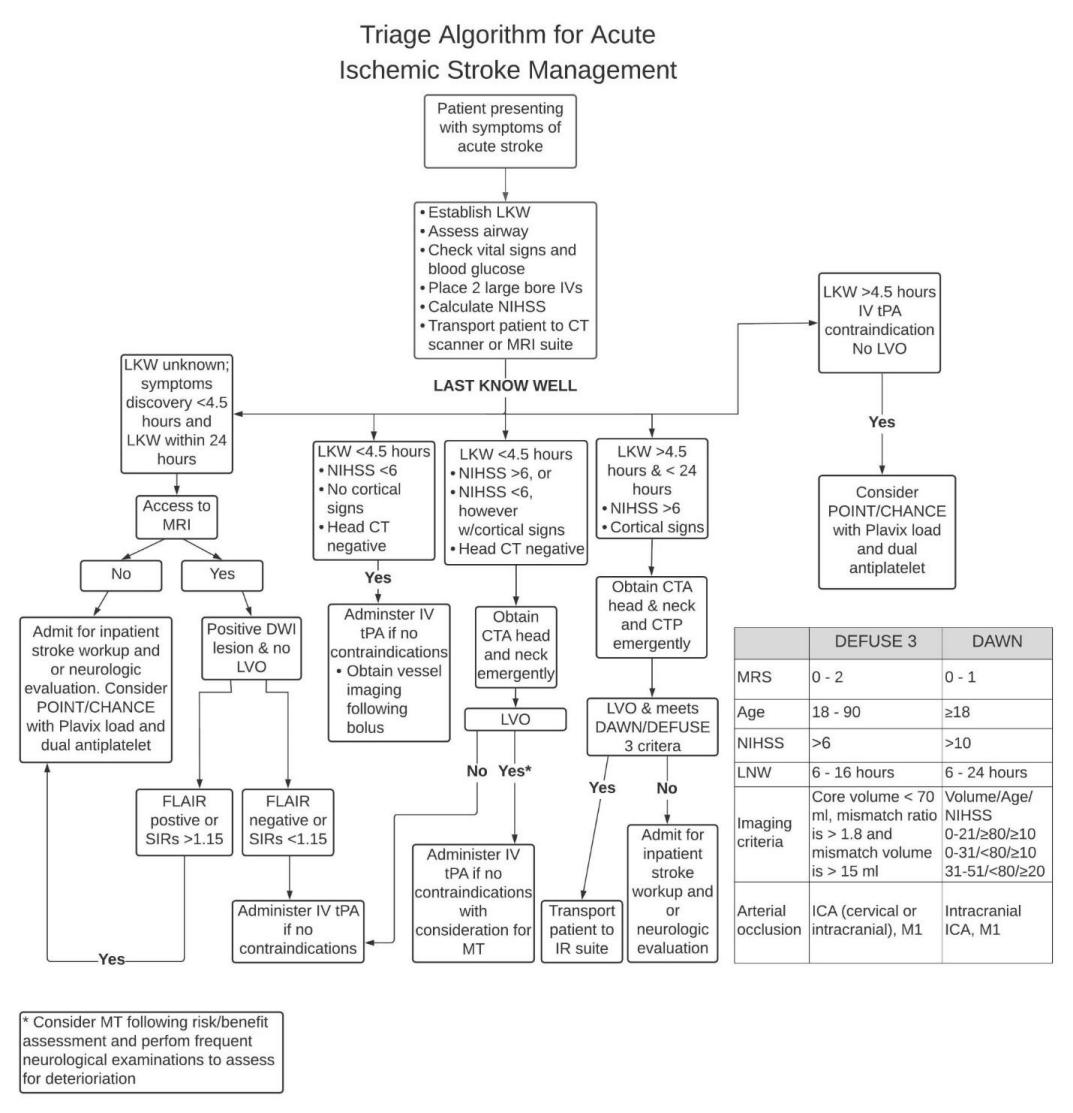
Algorithm for Acute Ischemic Stroke Management.

The CHANCE, POINT and THALES trials all provide evidence and further supports the role of dual antiplatelet therapy in stroke management. Table 3 provides a summary of the findings from these three trials. Optimal medical management with dual antiplatelet therapy in those who are not eligible for thrombolysis or endovascular therapy reduces the risk of early recurrent stroke. Although combination therapy is more effective, with all things considered, caution should be taken in regards to risk of hemorrhage. Triaging acute ischemic stroke involves a rapid, and concise approach to decision making. Figure 2 provides a pathway incorporating all treatment options: IV tPA, endovascular therapy with mechanical thrombectomy and dual antiplatelet therapy.

## 4.Conclusion

Since the publication of NINDS, several studies have made their way into literature, many of which have been discussed in this review. While there has been a lot of progress with the management of acute ischemic stroke, specifically, with extension of the time window for both MT and IV tPA, continued research is essential. It is important to not simply broaden the eligibility of patients who may benefit from our current repertoire of treatments, but to also identify specific cohorts of stroke patients who stand to benefit the most, especially with newer therapies. Future research must strive to establish efficacy and direct comparisons between novel treatments, and continue to push the boundaries of the care of acute ischemic stroke.

**Table 3:** Clinical Trials for Non-Treatment Candidates.

Study	Citation	Study Design	Intervention	Number of participants (n)	Rates of major/severe hemorrhage (Treatment versus Control)	Outcome
THALES	Johnston et al. NEJM 2020 [36]	Randomized, double-blind, placebo- controlled trial	Randomization within 24 hours of symptom onset	11,016	0.5% vs 0.1%, (P=0.001)	Lower risk of stroke or death within 30 days but increase risk of major hemorrhage.
POINT	Johnston et al. NEJM 2018 [25]	Randomized, double-blind multicenter trial	Randomization within 12 hours of symptom onset	4881	0.9% vs 0.4%, (P=0.02)	Lower risk of major ischemic events but a higher risk of major hemorrhage at 90 days
CHANCE	Wang et al. NEJM 2013 [24]	Randomized, double-blind, placebo- controlled trial	Randomization within 24 hours of symptom onset	5170	0.3% vs 0.3%, (P=0.73)	Lower risk of stroke but no increase risk of major hemorrhage at 90 days
